# Editorial: Virtual patients and digital twins in the systems analysis of drug discovery and development

**DOI:** 10.3389/fsysb.2023.1293076

**Published:** 2023-09-29

**Authors:** Chen Zhao, Hua He, Huilin Ma

**Affiliations:** ^1^ School of Pharmacy, Nanjing Medical University, Nanjing, China; ^2^ Center of Drug Metabolism and Pharmacokinetics, China Pharmaceutical University, Nanjing, China; ^3^ Bristol-Myers Squibb, Princeton, NJ, United States

**Keywords:** virtual patients, digital twins, quantitative systems pharmacology, drug development, computational modeling

## Introduction

In the past two decades, the concept of mechanistic physiological modeling has emerged and now become powerful tools in the modern drug development process, all the way from early preclinical target selection to late-stage clinical trials. As we gradually unveil the complex multi-dimensional nature of human diseases, how to use computational models to accurately reproduce the dynamical systems-level features and behaviors of diseases is now a central theme for ongoing research investigations. Mechanistic models that merge the essentials of systems biology and pharmacology (a good example of which is QSP, quantitative systems pharmacology) strive to include pathophysiological details across different scales (e.g., molecular-pathway-cellular-tissue-whole body) in order to simulate the progression and resolution of the disease upon pharmacological interventions, which can then provide cost-effective digital testing platforms to significantly reduce attrition and speed up translation. Through this Research Topic, we collected several recent efforts addressing this theme which are briefed below.

## Physiological model-based treatment simulation

Mechanistic modeling of disease pathophysiology can help reveal fundamental driving principles of the complex disease-drug interaction network, which can lead to the identification of potential new drug targets and new treatment strategies. Folkesson et al. developed a logical modeling framework that integrated signaling pathways frequently dysregulated in cancer as well as DNA damage-related features to explore potential new anti-cancer drug combinations. Using the colorectal cancer cell line HCT-116 as an example, high-throughput model simulations representing the decision-making of “a virtual cancer cell” correctly predicted the synergistic and nonsynergistic effects of third- and fourth-order drug combinations which are validated by experimental cell viability studies, highlighting the advantages of using computational modeling to efficiently rationalize drug testing and combination design. Another work by Santurio and Barros focused on the on-target off-tumor effects of CAR-T (chimeric-antigen receptor T) therapy, which has emerged as a promising new treatment modality for solid tumors including glioma. The authors formulated a mechanistic model based on the interaction between glioma pathophysiology and CAR-T cell action: growth and death of CAR-T cells, proliferation as well as CAR-T-mediated killing of tumor and healthy glial cells due to off-tumor target expression, and neuronal survival as a result of glial cell status. By assigning different combinations of physiological parameters to model-based virtual glioma patients, the authors analyzed the temporal kinetics of the above cells in sensitive *versus* resistant tumors and computationally examined the effect of different driving factors to propose new CAR-T dosing strategies that may potentially alleviate neuronal loss-related adverse effects for patients.

## Formulation of virtual patients and digital twins

Virtual patients and digital twins (these two terms are sometimes used interchangeably) represent the direct form of application of physiology-based computational models, especially in drug development scenarios. In this Research Topic, An and Cockrell described the definition of medical digital twins with a focus on how these model-based subjects and simulation projects can be methodologically formulated and utilized in typical drug development settings. They suggested that such models/digital twins should be as mechanistic as possible given the goal of simulating new therapeutic interventions, and they would require carefully designed validation protocols while providing enough capability of forecasting personalized treatment outcomes. On the other side, the review by Craig et al. presented a detailed walk-through of the key steps of how to deploy such model-based virtual patients and digital twins in the framework of virtual clinical trials. They also highlighted the importance of model sensitivity and identifiability analysis and how to balance these considerations in the creation of virtual patients with varying degrees of heterogeneity given different study purposes.

## Future perspectives

Although significant progress has been made in the conceptual and technical development of virtual patients and digital twins in simulating disease progression and clinical trials of new drugs and devices (Sové et al., 2020; Sarrami-Foroushani et al., 2021), challenges still remain for the more standardized and effective applications of these concepts in biomedical research and drug development. From the bottom-up perspective, considering the limited knowledge we already know compared to the vast unknowns of human biology, how to ensure the mechanistic correctness and comprehensiveness of any disease models we try to computationally formulate is of critical importance. Another key consideration to be resolved is how to embrace the explosive magnitude of multi-modal patient data in the age of precision medicine, and investigations in this direction can greatly enhance the predictive capacity of model-based virtual patients and digital twins. Therefore in the next decade, we envision that new techniques beyond traditional mechanism-based approaches, such as statistical modeling and machine learning, should be coupled together with systems biology/QSP models to collectively explore the above questions and pave the way to next level of precision medicine.
